# Effect of Technological Process and Temperature on Phospholipids in Buffalo Milk, Whey and Buttermilk

**DOI:** 10.3390/foods14152756

**Published:** 2025-08-07

**Authors:** Marika Di Paolo, Valeria Pelizzola, Lucia De Luca, Loriana Casalino, Giulia Polizzi, Milena Povolo, Raffaele Marrone

**Affiliations:** 1 Department of Veterinary Medicine and Animal Production, University of Napoli Federico II, 80137 Naples, Italy; marika.dipaolo@unina.it; 2 Research Centre for Animal Production and Aquaculture (CREA-ZA), Council for Agricultural Research and Economics, 26900 Lodi, Italy; valeria.pelizzola@crea.gov.it (V.P.); milena.povolo@crea.gov.it (M.P.); 3 Department of Agricultural Sciences, University of Napoli Federico II, 80138 Naples, Italy; lucia.deluca@unina.it; 4 Department of Economic and Legal Sciences, Universitas Mercatorum, 00186 Rome, Italy; loriana.casalino@studenti.unimercatorum.it; 5 Department of Veterinary Medical Sciences, University of Bologna, Via Tolara di Sopra, 50, 40064 Ozzano dell’Emilia, Italy; giulia.polizzi3@unibo.it

**Keywords:** technological processing, by-products, HPLC/ELSD, thermal process

## Abstract

Phospholipids (PLs) are a group of biomolecules found in the milk fat globule membranes (MFGMs). Recently, MFGM phospholipids have attracted increasing amounts of attention due to their unique composition, stability, and potential health benefits, including protective effects against Alzheimer’s disease, hypercholesterolemia, and certain types of cancer. Although buffalo milk is the second most commonly produced milk and has high nutritional value, few studies have focused on the properties of buffalo MFGM. This study investigates the PLs composition of buffalo milk and related dairy by-products (whey and buttermilk). Milk and whey were collected from two dairy farms (A—small and B—big) to produce mozzarella buffalo cheese (high-pasteurization milk for GDO production and low for local); while buttermilk was obtained from a butter-making farm. Phospholipids were purified by a solid-phase extraction method and then identified by high-performance liquid chromatography with an evaporative light-scattering detector (HPLC/ELSD). Five classes of phospholipids [phosphatidylcholine (PC), phosphatidylethanolamine (PE), phosphatidylinositol (PI), phosphatidylserine (PS), and sphingomyelin (SM)] were identified. The thermal process of milk did not significantly affect the PLs milk. However, local whey showed a higher concentration of total PLs than GDO, which was mainly represented by PE followed by PC content. Farm A exhibited higher PL content than B, particularly with a greater concentration of SM. Buttermilk showed the lowest PLs content. These findings offer valuable insights for the dairy industry and related applications, contributing to the valorization of buffalo dairy products.

## 1. Introduction

Phospholipids (PLs) are a class of biomolecules present in the milk fat globule membranes (MFGM), which accounts for approximately 60–70% of the total phospholipids (PLs) in milk. These compounds constitute about 0.5–1% of the total milk lipids and are primarily composed of sphingolipids and glycerophospholipids, both characterized by their polar nature. As amphiphilic molecules, PLs consist of hydrophobic fatty acyl chains and hydrophilic organophosphate head groups, such as choline, serine, inositol, or ethanolamine [1]. Recently, attention has been drawn to MFGM phospholipids due to their composition, stability, and potential health benefits, such as their ability to suppress Alzheimer’s disease and protect against hypercholesterolemia and cancer [2]. Moreover, dairy PLs are recognized for their excellent emulsifying properties, which are attributed to their amphiphilic nature. For this reason, they can be used as an alternative to industrial lecithin for stabilizing emulsions in foods [3]. Milk and dairy by-products, such as butter serum, whey, and buttermilk, are natural sources of PLs [4,5]. Numerous studies have investigated the distribution of phospholipids in bovine milk [1,6], as well as in the milk of other species, such as goat and ewe [7]. However, only a limited number of studies on buffalo milk and its by-products are currently available. The existing literature is largely composed of review articles that primarily focus on the compositional characteristics, as well as the health-promoting and functional properties of buffalo milk and whey derived from cheese production. Considering that buffalo milk accounts for approximately 15% of global milk production and that both buffalo milk and cheese production are increasing [8], the buffalo dairy sector represents a high-volume industry in terms of milk and by-products [9]. These by-products hold significant potential for valorization, which could enhance profitability through added value from processing and contribute to reducing the environmental footprint of the dairy industry. Among dairy by-products, buttermilk, which is produced during the churning of cream into butter, has attracted considerable interest due to its PLs content. During butter production, the MFGM, which is rich in PLs, is disrupted and partially released into the buttermilk, making it a potential source of these compounds [10]. Similarly, whey from mozzarella cheese production also appears to be a promising source of PLs, primarily composed of phosphatidylcholine (PC), phosphatidylethanolamine (PE), and sphingomyelin (SM), while phosphatidylserine (PS) and phosphatidylinositol (PI) are present in smaller amounts [11].

The characteristics of milk and the variation in milk components are affected not only by the characteristics of the raw milk but also by the technological processes applied, such as heat treatments [12] and homogenization [13]. In fact, since PLs are mainly found in MFGMs, their composition in dairy products (final products and by-products) is affected by any treatment that causes a perturbation of the membrane and/or a separation or fractionation of fat globules, such as homogenization and/or centrifugation of polar and neutral components of fat. Thermal treatments, in particular, may compromise the structural integrity of the MFGM, leading to partial loss or redistribution of PLs across the serum and fat phases [7]. However, milder processes such as low-temperature pasteurization can help preserve these compounds, enhancing the nutritional value of the final products and by-products. Considering the nutritional and functional properties of PLs, there is a growing interest in developing methods for their extraction and concentration from dairy by-products [14]. A new strategy to concentrate PL by-products using food-grade green methods based on ethanol-modified supercritical carbon dioxide (SC-CO2) extraction has been developed [15,16]. Others have described pilot-scale production of a phospholipid-enriched dairy ingredient using an optimized integrated process employing enzymatic hydrolysis, ultrafiltration, and supercritical fluid extraction [17]. The choice of processing techniques plays a critical role not only in determining the yield of phospholipid recovery, but also in preserving the bioactive potential of these molecules. Innovations in green extraction technologies further support the sustainable development of high-value ingredients from dairy streams.

In this regard, the economic potential of valorizing by-products is evident. The scientific literature has highlighted new properties and proposed broader uses for buttermilk and MFGM isolates in a variety of food products, including evaporated milk, yogurt, cheddar cheese, mozzarella, pizza cheese, and low-fat cheese [4]. Recently, the supercritical fluid extraction (SFE) technique has gained significant attention in the food industry for extracting phospholipids (PLs) [18]. By using supercritical solvents, SFE offers high selectivity for isolating natural components from complex matrices, making it a promising and environmentally friendly food-processing method for efficiently extracting nonpolar lipid compounds. Nevertheless, despite this growing interest, buffalo milk and its by-products remain underexplored in terms of phospholipid profiling, particularly considering their unique compositional characteristics. A detailed analysis of the effects of dairy processing on buffalo PL content and distribution is therefore necessary to unlock the full potential of these matrices. To evaluate the potential value of buffalo milk by-products, and considering the lack of studies on their characterization, it is essential to investigate their properties to assess their potential applications. In this context, the aim of the study was to analyze the phospholipid composition of milk and by-products from buffalo mozzarella and butter production.

## 2. Materials and Methods

### 2.1. Sampling

Buffalo milk and whey samples were collected from two dairies (dairy A, a small self-supplying dairy; and dairy B, a big dairy with 43 milk suppliers) in Caserta province (Campania Region, Italy). Buttermilk samples were collected from a butter-making farm in Napoli province (Campania Region, Italy).

For each dairy, two different mozzarella cheese-making processes were considered: a process intended to facilitate large-scale distribution, in which the milk was pasteurized at 75 °C for 15 s (GDO process); and a process intended for local sales, in which the milk was heated at 64 °C for 15 s (LOC process). In a butter-producing farm, buttermilk was collected after the cream underwent pasteurization, ripening, and churning.

A total of 24 samples of milk and whey were collected. For each dairy, for the LOC production process, three samples of thermized buffalo milk (milk LOC) and three samples of buffalo whey obtained at the end of the LOC process (whey LOC) were collected. Similarly, for the GDO production process, three samples of pasteurized buffalo milk (milk GDO) and three samples of buffalo whey obtained at the end of the GDO process (whey GDO) were collected. Additionally, three buttermilk samples obtained from butter production were collected.

### 2.2. Compositional Analyses

Milk, whey, and buttermilks were analyzed to determine their total protein [19] and total fat content. The total lipids were extracted from samples following the procedure reported by Folch et al., 1957 [20]. Briefly, the samples were dissolved in 1000 mL chloroform–methanol (2:1, *v*/*v*) mixture. The organic phase containing lipid samples was collected and equilibrated with ¼ volume of a saline solution (NaCl 0.05 N). The lower chloroform layer was filtered and evaporated under a vacuum, and the obtained total lipids were stored at −20 °C until further analysis.

### 2.3. Phospholipids (PLs) Purification

PLs were separated from neutral lipids (NLs) by solid-phase extraction (SPE), by applying the conditions reported by Contarini et al. [19]. The lipid sample (200 mg) was dissolved in 0.5 mL of n-hexane and then the fat solution was loaded on the SPE cartridge DSC-Si 6 mL, 1 g. After conditioning with n-hexane, the nonpolar lipids were eluted with 10 mL of n-hexane–diethyl ether (1:1, *v*/*v*). The recovery of phospholipids was performed by using two different conditions: the first with 10 mL of methanol and the second with 10 mL of methanol–chloroform–water (5:3:2, *v*/*v*/*v*). The PL fraction was dried under a gentle stream of nitrogen.

### 2.4. Phospholipid Analysis by HPLC-ELSD

The dried PL fraction was re-dissolved in 600 µL of chloroform–methanol (80:20, *v*/*v*) and analyzed using a Shimadzu HPLC system (Kyoto, Japan). The system was equipped with two LC-10 Advp pumps, an SCL-10 Advp gradient system, a DGU-14 Advp degasser module, and an automatic injector. A silica normal-phase column (150 mm × 3 mm, I.D. 3 µm) Reprosil 100 Si (Dr. Maisch HPLC GmbH, Ammerbuch-Entringen, Germany) was employed, along with a Sedex Model 75 evaporative light-scattering detector (ELSD) (S.E.D.E.R.E., Alfortville, France) for PL separation and detection. Chromatographic separation followed the conditions described by Contarini et al. [19]. The mobile phases consisted of dichloromethane (eluent A) and a methanol–acetic acid/triethylamine (2:1)–water mixture (50:1:1, *v*/*v*/*v*) as eluent B. A linear binary gradient was applied as follows: 0 min, 96% A; 4 min, 88% A; 17 min, 94% B; 21 min, 96% A, held for 7 min. The eluent flow rate was maintained at 0.5 mL min^−1^. For identification and calibration, pure PL standards (PI, PE, PS, PC, and SM) were purchased from Lab Service Analytica S.r.l. (Anzola dell’Emilia, BO, Italy). All the reagents for PL analysis were of HPLC-grade. PLs were identified by comparing retention times with those of pure standards, and calibration curves were prepared for each compound. All analyses were performed in triplicate, and the results were expressed as milligrams per 100 g of fat (mg/100 g fat). In addition, the relative proportion of polar lipids was calculated and expressed as a percentage of total polar lipids.

### 2.5. Statistical Analysis

Variance analyses (ANOVA) were conducted using the General Linear Model procedure in Statgraphics Plus, Version 5 (Statistical Graphics Corp., Englewood Cliffs, NJ, USA). Treatment means were compared using Fisher’s least significant difference (LSD) test, with a significance level set at 5%. Spearman’s correlation analyses were conducted to assess the relationship between processing temperature and the phospholipid (PL) content in buffalo milk and whey. The analysis was performed using the values of PL (*n* = 3) for each treatment condition (64 °C and 75 °C), considering both matrices (milk and whey) and dairy sources (A and B). Individual PL classes (phosphatidylethanolamine, phosphatidylcholine, phosphatidylserine, phosphatidylinositol, and sphingomyelin) as well as total PL content were considered. The statistical significance was set at *p* < 0.05.

## 3. Results and Discussion

### 3.1. Chemical Composition

Buffalo milk is a nutritionally rich raw material that is widely used in dairy processing. During the production of cheese and butter, by-products such as whey and buttermilk are obtained. Although traditionally considered to be waste products, these by-products exhibit nutritional and functional value. Characterizing their composition is essential to demonstrate their nutritional value and explore opportunities for their valorization. The chemical composition of buffalo milk, whey, and buttermilk is shown in Table 1. As expected, buffalo milk exhibited the highest concentrations of fat and protein, with fat content ranging from 7.40% to 7.95% and protein content from 4.39% to 4.47%. Notably, significant differences were found in fat content, with dairy B showing the highest levels. Overall, the values for protein and fat are consistent with the results of previous research [21]. Buffalo whey, obtained after the production of buffalo mozzarella, showed a marked reduction in fat content, ranging from 0.14% to 0.22%, and protein levels between 1.06% and 1.28%. These results indicate that most of the fat is utilized during cheese-making. At the same time, the buttermilk showed a low fat concentration, indicating that most of the fat content was used during butter production. Moreover, the buttermilk demonstrated the lowest protein concentrations, with values of 0.82%. No differences were observed based on different temperature processes. These findings underscore the variability in nutritional composition at different stages of the technological process.

### 3.2. Identification and Composition of Phospholipids

Phospholipids (PLs) are essential components of the milk fat globule membrane (MFGM), playing a crucial role in membrane structure, cellular signaling, and lipid metabolism. Their amphiphilic nature contributes not only to the stability of fat globules but also to various biological functions, including anti-inflammatory activity, neuroprotection, and immune modulation [1]. The identification and quantification of PLs in milk and its by-products provide key insights into the nutritional quality and potential health-promoting properties of these matrices. The concentration and relative portion of polar lipids in the fat globule membrane of buffalo milk and whey is shown in Table 2 and Table 3. Five classes of phospholipids (PLs) were identified in the samples, including phosphatidylcholine (PC), phosphatidylethanolamine (PE), phosphatidylinositol (PI), phosphatidylserine (PS), and sphingomyelin (SM). The most abundant classes were PE and PC, as also described by Lee et al. [22] and Wang et al. [23]. Notably, phosphatidylcholine (PC) is one of the few compounds able to cross the blood–brain barrier, allowing it to reach brain cells directly. There, it is utilized to produce acetylcholine (ACh), a neurotransmitter that may enhance memory [24]. According to Roy et al. [24], exogenous PC participates in anti-inflammatory signaling pathways and exhibits immune-modulatory effects. Furthermore, the intake of exogenous PC has been shown to strengthen intestinal barrier defense by inhibiting the production of pro-inflammatory cytokines [25]. Overall, samples from dairy A exhibited higher total PL content (mg/100 g of fat) compared to those from dairy B, with a particularly higher SM concentration in dairy A. This finding is significant due to the protective role of SM against colon cancer [26].

#### 3.2.1. Influence of Temperature on PLs Profile

Thermal treatments can significantly impact the integrity of the milk fat globule membrane (MFGM), potentially altering the structure, distribution, and concentration of its associated polar lipids. While pasteurization and other heating methods are essential for microbiological safety and shelf-life extension, they may induce changes in membrane fluidity, protein–lipid interactions, and phospholipid (PL) composition [1,7]. These modifications can lead to the redistribution or partial degradation of MFGM components, especially under high-temperature conditions. Understanding how different thermal processes affect the PL profile of milk and its derivatives is critical, given the nutritional and functional importance of these compounds [27]. Based on our study, low-temperature treatments at 64 °C for 15 s (LOC process) are believed to preserve MFGM integrity more effectively, limiting phospholipid loss and maintaining the bioactive properties of milk fat globules. The results indicated that the thermal processing of milk has a minimal effect on its total phospholipid (PL) content. In fact, in milk processed using the LOC method, the total PL content was 78.59 mg/100 g in dairy A and 58.31 mg/100 g in dairy B, compared to 69.56 mg/100 g in dairy A and 80.98 mg/100 g in dairy B for the GDO method. However, whey obtained through the LOC process exhibited a higher total PL concentration than that from the GDO process, with PE and PC being the predominant classes in both dairy industries (A and B). Despite these differences, no significant correlation was observed between temperature and individual phospholipids (PI, PE, PS, PC, SM), nor with the total PL content (ρ ranging from –0.218 to 0.000, *p* > 0.05). These results are in line with findings by Argov-Argaman et al. [7], who demonstrated that heat treatment, such as pasteurization at 76 °C for 15 s, can affect phospholipid composition. These polar lipids are bioactive compounds that play a crucial role in defining the structural properties of membranes and lipoproteins, contributing to the notable nutritional value of buffalo milk [5,27]. The LOC process, characterized by lower temperatures, appears to better preserve PLs compared to the GDO process. In particular, the low-temperature treatment limits PL loss, which would otherwise redistribute into other fractions during high-temperature processing. In whey samples, the LOC process yielded a total PL concentration of 2587.03 mg/100 g in dairy A and 1909.95 mg/100 g in dairy B, nearly double that of the GDO process (1654.90 mg/100 g in dairy A and 1311.04 mg/100 g in dairy B). Among the individual PL classes, PE and PC were predominant in the LOC whey, with a concentration of 836.93 and 727.91 mg/100 g in dairy A, and 594.71 and 517.76 in dairy B, respectively.

Interestingly, the total PL content in milk of the dairy B was higher in GDO samples (80.98 mg/100 g) than in LOC samples (58.31 mg/100 g). Although the differences between GDO and LOC observed in the milk of dairy A were not replicated in dairy B, it is important to note that these differences were not statistically significant. This is likely due to the high variability in the milk from dairy B, which can be attributed to the larger size of the company and the greater number of milk suppliers, resulting in increased heterogeneity of the raw material [28]. In contrast, whey from LOC samples of dairy B had a higher concentration of PLs (1909.95 mg/100 g) compared to GDO samples (1311.04 mg/100 g), with PE and PC again being the dominant classes.

#### 3.2.2. Influence of Technological Processes

The overall technological process applied to milk, including separation, homogenization, churning, and curdling, can significantly influence the structure and composition of the milk fat globule membrane (MFGM). These mechanical and biochemical steps induce a redistribution of membrane components, particularly polar lipids, among the cream phase, serum, and residual matrices such as whey and buttermilk [2,29]. The reorganization of phospholipids reflects the complex dynamics of membrane destabilization during processing. Investigating these shifts is crucial to better understand the fate of bioactive lipids throughout the dairy production chain and to optimize the recovery and potential valorization of nutritionally valuable by-products.

Milk-processing methods influence the composition of PLs in the milk fat globule membrane (MFGM), causing a reorganization between the membrane, milk serum, and other fractions, according to Sun et al. [29]. It was observed that the content of PC was affected by milk processing, showing an increase of approximately 6% in whey samples compared to milk regardless of the thermal treatment, according to Ménard et al. [30]. In particular, the percentage of phosphatidylcholine (PC) detected in samples from dairy A increased from 22.08% and 22.59% in LOC and GDO milk, respectively, to 29.39% and 28.77% in LOC and GDO whey, respectively (Table 3). The percentage of PC detected in samples from dairy B increased from 20.74% and 20.80% in LOC and GDO milk, respectively, to 27.01% and 26.65% in LOC and GDO whey, respectively (Table 3).

Contrary to Thum et al. [28] buttermilk showed the lowest polar lipid (PL) content among the analyzed samples, although this difference was not statistically significant (Table 4). Even though buttermilk showed a higher fat content than whey (Table 1) and is produced in significantly larger volumes, this by-product remains largely underutilized [31]. Considering the fat balance, a substantial portion of milk fat is retained in buttermilk after butter production, making it a rich source of valuable lipids. In contrast, whey is already partially used in the production of dairy products such as ricotta and is recognized as a valuable source of bioactive peptides [32]. The underutilization of buttermilk, combined with its favorable fat composition, highlights its potential as an excellent source for developing functionalized ingredients. Therefore, buttermilk represents a promising opportunity to add value to what is often regarded as a low-value by-product, contributing to more effective waste valorization within the dairy industry.

## 4. Conclusions

This study provides novel insights into the phospholipid (PL) profile of buffalo milk and its by-products, highlighting the impact of technological processes and thermal treatments on their composition. Our results show that while the thermal treatment of milk does not significantly alter its PL content, lower-temperature processes (LOCs) are more effective at preserving phospholipids in whey. Among the by-products, whey obtained from LOC emerged as the richest source of phospholipids, particularly PE and PC, reinforcing its value as a functional ingredient. Conversely, buttermilk—despite showing the lowest overall PL content—retained considerable potential for phospholipid recovery due to its fat composition and availability as an underutilized by-product. The compositional differences observed between dairies further suggest that milk quality and process standardization may also influence the distribution and yield of PLs, opening up opportunities for tailored strategies in dairy valorization. These results underscore the importance of optimizing dairy-processing techniques to maximize the retention and recovery of bioactive compounds such as phospholipids. Integrating such optimization into existing dairy chains could improve process efficiency, reduce waste, and deliver high-value functional ingredients for food, nutraceutical, and pharmaceutical applications. This approach not only improves the nutritional quality of dairy-derived ingredients but also supports the circular economy by enhancing the value of by-products. Future research should focus on scaling up efficient extraction methods and exploring industrial applications of PL-enriched ingredients, particularly from buffalo milk, to foster innovation and sustainability in the dairy sector.

## Figures and Tables

**Table 1:** Chemical analysis of buffalo milk, whey and buttermilk (mean ± standard deviation). GDO, pasteurized milk 75 °C for 15 s; LOC, thermized milk 64 °C for 15 s.

			Fat%	Protein%
Buffalo milk	LOC	A	7.60 ± 0.03 ^a,x^	4.45 ± 0.12
		B	7.95 ± 0.10 ^b^	4.47 ± 0.22
	GDO	A	7.40 ± 0.07 ^a,y^	4.39 ± 0.04
		B	7.90 ± 0.12 ^b^	4.40 ± 0.09
Buffalo whey	LOC	A	0.16 ± 0.04	1.28 ± 0.03
		B	0.14 ± 0.08 ^x^	1.23 ± 0.04
	GDO	A	0.22 ± 0.13	1.28 ± 0.01
		B	0.22 ± 0.09 ^y^	1.06 ± 0.16
Buttermilk	-	-	0.33 ± 0.09	0.82 ± 0.08

A—a small dairy self-supplying the dairy industry and B—a big dairy industry with 43 milk suppliers. ^a–b^ Different superscript lowercase letters indicate a significant difference at *p* < 0.05. ^a–b^ Mean values between A and B dairy industry with different letters presented significant differences. ^x–y^ Mean values between LOC and GDO process with different letters presented significant differences.

**Table 2:** Concentration of polar lipids (mg/100 g of fat) in the fat globule membrane of buffalo milk and whey (mean ± standard deviation) in dairy A and B.

Concentration in Polar Lipid (mg/100 g of Fat)					
		Buffalo Milk		Buffalo Whey	
Items	Dairy	LOC	GDO	LOC	GDO
PI	A	6.34 ± 1.29	5.94 ± 0.09	224.77 ± 6.14	121.00 ± 8.53
	B	7.39 ± 1.33	8.43 ± 0.97	147.43 ± 10.77	99.99 ± 6.33
PE	A	33.06 ± 19.83	26.01 ± 6.99	836.93 ± 66.53	453.41 ± 16.50
	B	18.62 ± 0.64	27.13 ± 9.19	594.71 ± 122.40	341.51 ± 88.30
PS	A	7.62 ± 0.26	7.23 ± 0.04	362.60 ± 4.46	305.82 ± 2.54
	B	9.18 ± 1.79	13.77 ± 2.97	268.33 ± 42.55	275.34 ± 43.89
PC	A	17.62 ± 7.61	15.71 ± 4.82	727.91 ± 38.08	465.42 ± 15.66
	B	12.55 ± 3.34	16.99 ± 6.68	517.76 ± 97.55	340.67 ± 34.38
SM	A	13.95 ± 4.76	14.67 ± 6.43	434.81 ± 49.54	309.25 ± 14.45
	B	10.56 ± 5.40	14.66 ± 10.23	381.70 ± 22.79	253.54 ± 12.09
Polar lipids	A	78.59 ± 30.61	69.56 ± 18.11	2587.03 ± 155.83	1654.90 ± 52.62
	B	58.31 ± 17.37	80.98 ± 27.61	1909.95 ± 264.54	1311.04 ± 155.76

A—small dairy self-supplying the dairy industry and B—a big dairy industry with 43 milk suppliers. PC—phosphatidylcholine; PE—phosphatidylethanolamine; PI—phosphatidylinositol; PS—phosphatidylserine and SM—sphingomyelin.

**Table 3:** Relative proportion of polar lipids (% of polar lipids) in the fat globule membrane of buffalo milk and whey (mean ± standard deviation) in dairy A and B.

Relative Proportion of Polar Lipids (% of Polar Lipids)					
		Buffalo Milk		Buffalo Whey	
Items	Dairy	LOC	GDO	LOC	GDO
PI	A	8.58 ± 2.02	8.54 ± 2.44	9.07 ± 0.24	7.48 ± 0.53
	B	12.74 ± 0.84	10.95 ± 2.55	7.83 ± 1.64	7.67 ± 0.54
PE	A	39.75 ± 9.53	37.39 ± 0.32	33.79 ± 2.68	28.03 ± 1.02
	B	32.79 ± 6.95	33.51 ± 2.92	31.02 ± 1.58	25.76 ± 4.01
PS	A	11.03 ± 5.25	10.39 ± 2.86	14.64 ± 0.18	18.90 ± 0.16
	B	16.93 ± 1.22	17.77 ± 4.81	14.03 ± 0.28	21.07 ± 2.87
PC	A	22.08 ± 1.58	22.59 ± 1.09	29.39 ± 1.54	28.77 ± 0.97
	B	20.74 ± 1.48	20.80 ± 1.55	27.01 ± 1.36	26.03 ± 0.65
SM	A	18.57 ± 5.19	21.08 ± 3.89	17.56 ± 2.00	19.12 ± 0.89
	B	16.79 ± 3.39	16.97 ± 6.43	20.09 ± 1.59	19.47 ± 1.80

A—small dairy self-supplying the dairy industry and B—a big dairy industry with 43 milk suppliers. PC—phosphatidylcholine; PE—phosphatidylethanolamine; PI—phosphatidylinositol; PS—phosphatidylserine; and SM—sphingomyelin.

**Table 4:** Concentration of polar lipids (mg/100 g of fat) in the fat globule membrane of buffalo by-products such as LOC whey (dairy A and B) and buttermilk (mean ± standard deviation).

Concentration in Polar Lipid (mg/100 g of Fat)			
Items	Whey A	Whey B	Buttermilk
PI	224.77 ± 6.14	147.43 ± 10.77	73.51 ± 8.63
PE	836.93 ± 66.53	594.71 ± 122.40	412.26 ± 100.97
PS	362.60 ± 4.46	268.33 ± 42.55	163.97 ± 47.69
PC	727.91 ± 38.08	517.76 ± 97.55	309.56 ± 52.99
SM	434.81 ± 49.54	381.70 ± 22.79	253.50 ± 44.38
Polar lipids	2587.03 ± 155.83	1909.95 ± 264.54	1212.80 ± 232.87

A—a small dairy self-supplying the dairy industry and B—a big dairy industry with 43 milk suppliers. PC—phosphatidylcholine; PE—phosphatidylethanolamine; PI—phosphatidylinositol; PS—phosphatidylserine; and SM—sphingomyelin.

## Data Availability

The original contributions presented in this study are included in the article. Further inquiries can be directed to the corresponding author.
